# Limited Contribution of DNA Methylation Variation to Expression Regulation in *Arabidopsis thaliana*

**DOI:** 10.1371/journal.pgen.1006141

**Published:** 2016-07-11

**Authors:** Dazhe Meng, Manu Dubin, Pei Zhang, Edward J. Osborne, Oliver Stegle, Richard M. Clark, Magnus Nordborg

**Affiliations:** 1 Gregor Mendel Institute, Austrian Academy of Sciences, Vienna Biocenter, Vienna, Austria; 2 Molecular and Computational Biology, University of Southern California, Los Angeles, California, United States; 3 Department of Biology, University of Utah, Salt Lake City, United States; 4 European Molecular Biology Laboratory, European Bioinformatics Institute, Wellcome Trust Genome Campus, Hinxton, Cambridge, United Kingdom; University of California Irvine, UNITED STATES

## Abstract

The extent to which epigenetic variation affects complex traits in natural populations is not known. We addressed this question using transcriptome and DNA methylation data from a sample of 135 sequenced *A. thaliana* accessions. Across individuals, expression was significantly associated with *cis*-methylation for hundreds of genes, and many of these associations remained significant after taking SNP effects into account. The pattern of correlations differed markedly between gene body methylation and transposable element methylation. The former was usually positively correlated with expression, and the latter usually negatively correlated, although exceptions were found in both cases. Finally, we developed graphical models of causality that adapt to a sample with heavy population structure, and used them to show that while methylation appears to affect gene expression more often than expression affects methylation, there is also strong support for both being independently controlled. In conclusion, although we find clear evidence for epigenetic regulation, both the number of loci affected and the magnitude of the effects appear to be small compared to the effect of SNPs.

## Author Summary

It has been demonstrated experimentally that epigenetic variation, in particular DNA methylation, can transmit information across generations. However, it is difficult to evaluate the importance of such effects in natural populations due to complex genetic background effects, making experimental the separation of genetic and epigenetic effects challenging. Here we use quantitative genetic models to test whether epigenetic variation plays a significant role in gene expression variation once genetic variation has been taken into account. In addition, we devise and apply methods that go beyond a simple association framework in order to infer causal relationships. Our results suggest a significant but small epigenetic contribution to expression regulation.

## Introduction

It has been long speculated that epigenetic modifications, in particular DNA methylation, contribute to heritable phenotypic variation [1, 2]. That the potential exists is not in doubt, especially in plants. Modern sequencing technology allows us to investigate DNA methylation on a genomewide scale, and has revealed that spontaneous changes in DNA methylation, or epimutations, can be inherited without accompanying DNA changes [3, 4], and that induced DNA methylation changes in genetically homogeneous lines can bring about heritable phenotypic changes [5].

However, these studies tell us nothing about the importance of epigenetic inheritance relative to actual genetic variation, which is typically substantial in natural populations. Recent population studies in *A. thaliana* have suggested a role for DNA methylation [6, 7], but did not explicitly investigate DNA methylation effects on top of SNP effects. To further address this question, we utilized an existing data set comprising genome-, epigenome-, and transcriptome-sequencing data for a population of 135 Swedish *A. thaliana* accessions [7].

We consider two types of DNA methylation: C methylation (or TE-like methylation) and CG-only methylation (or gene body methylation), defined as in previous work [7]. The former is characterized by heavy methylation in all contexts (CG as well as non-CG), involves the pathways dependent on RNA-directed DNA methylation (RdDM) or *CMT2* [8, 9], and is associated with heterochromatin and the silencing of mobile elements [10]. The latter involves sparse CG methylation of a subset of “housekeeping” genes; its presence and level is evolutionarily conserved [11] and it is generally positively correlated with transcription.

Based on type distinction and DNA context of the methylated cytosine, we divided DNA methylation variants into four non-overlapping sets: CG where no non-CG methylation is present; CG where there is non-CG methylation present; CHG, and, finally; CHH methylation. These variants are quantified by averaging methylation level of cytosines over all eligible cytosines in 200 bp windows (see Methods). As the four types have different baseline levels and involve different pathways, we normalized their levels and performed most analysis separately.

Our study faces statistical challenges in terms of strong population structure, which not only leads to the usual difficulties for genome-wide association studies [12], but also means that DNA methylation variation will be strongly correlated with DNA variation due to linkage disequilibrium as well as direct causation [7]. In what follows we present several novel mixed-model methods that aim to solve these problems.

## Results

### Expression and *cis*-methylation

**Fig 1:**
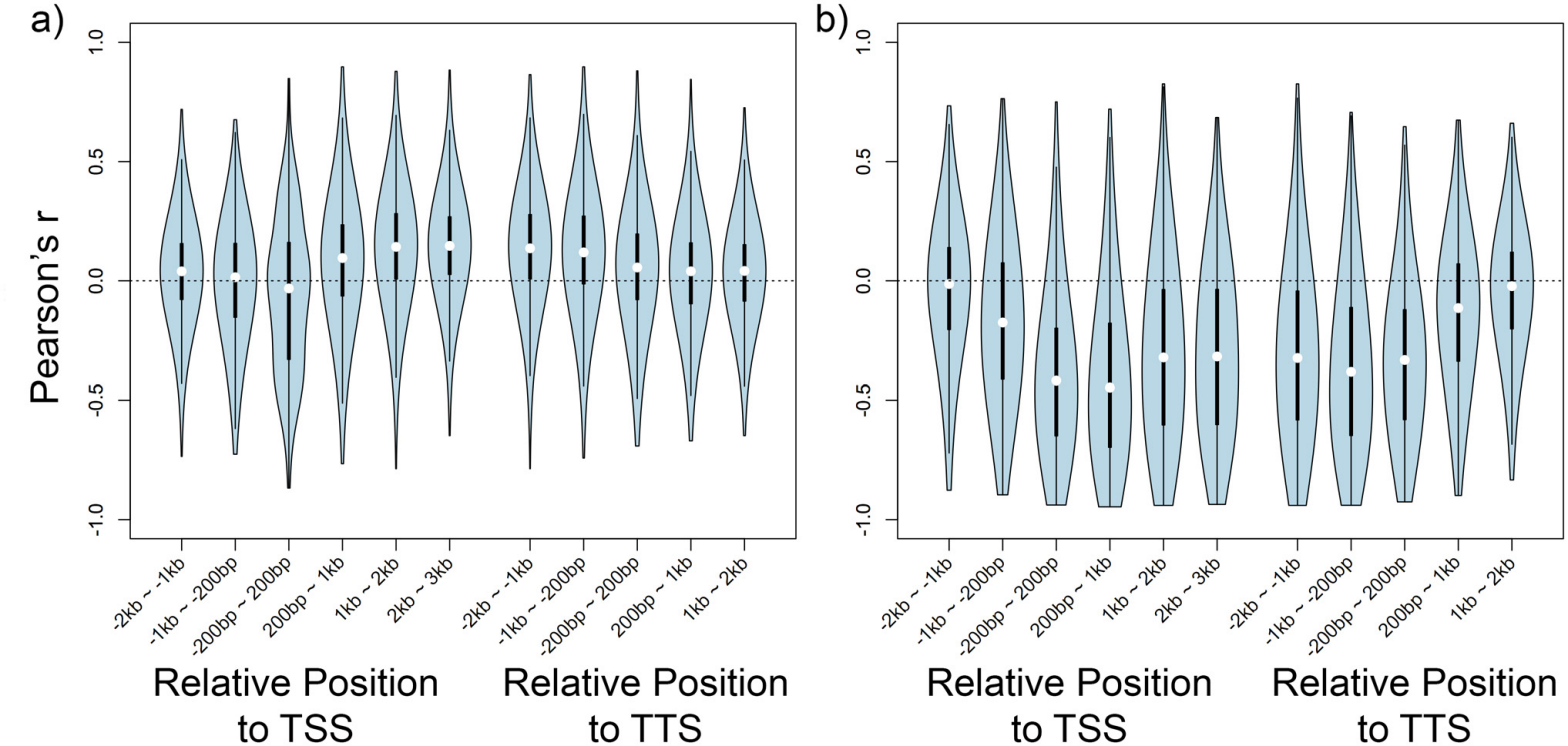
Correlation between DNA methylation level and expression across individuals. Correlation is shown with violin plots as distribution of Pearson’s *r* along genes. a) Genes most strongly associated with a CG-only methylation variant. b) Genes most strongly associated with a C methylation variant. TSS and TTS are transcription start sites and transcription termination sites, respectively.

Several studies have investigated correlation between gene expression and local DNA methylation across genes within a single or a small number of genetic backgrounds [13, 14]. Here, we investigate correlations between gene expression and local DNA methylation across many individuals (with distinct genetic backgrounds) for each gene instead. An immediate conclusion is that the relationship between DNA methylation and expression is not simple, but generally agrees with previously published results [15–20]. While CG-only methylation typically shows a weak positive correlation with expression, it can also be negatively correlated, and while C methylation generally shows a strong negative correlation with expression, it can also be positively correlated (Fig 1). Similar variation is found if we consider the pattern of correlations along genes. For genes with CG-only methylation, there is a clear tendency towards positive correlations in the middle of genes, whereas for genes with C methylation, strong negative correlations are found at the transcription start and termination sites.

### Expression and genome-wide methylation

If phenotypic variation is due to many polymorphisms of small effect, we expect a linear relationship between genetic relatedness and phenotypic covariance [21]. While relatedness was historically estimated from pedigrees, genome-wide SNP data make it possible to estimate it directly, and this fact has recently been exploited to estimate the fraction of phenotypic variation that is attributable to genetic relatedness, i.e., is due to genetic variation [22]. The same approach can also be used to control for the genetic background in GWAS [12, 23, 24], and to further attribute genetic contributions to specific chromosomes [25], annotation units [26], or even loci [27]. We applied the same technique to epigenetic markers, and asked the question whether genome-wide similarity in DNA methylation helps explains expression variation. Formally, we seek to compare a genome-wide small effects model that includes only SNPs to models that also includes methylation (see Methods). We considered CG-only and C methylation separately and together, but the results were unaffected by this.

When comparing models that include methylation as well as SNPs to a model that does not, only 261 genes show marginally significant effects, and none are significant after taking multiple testing into account. Thus, including genome-wide methylation as a background effect did not explain any additional variation in gene expression. This does not mean that background methylation has no effect, because methylation variation is highly correlated with SNP variation (either due to linkage, or direct causation [7]), and identifying a separate, orthogonal effect statistically may be very difficult. It does mean that there is no reason to include methylation as well as SNPs when correcting for background effects.

Out of curiosity, we can also performed the reverse analysis: do we need SNPs if we have methylation? The answer is similar (455 genes showed marginally significant effects of SNPs once methylation was taken into account), again emphasizing the very strong correlation between genetic variation and DNA methylation.

### Genome-wide association scans

Although genome-wide methylation relatedness does not help explain phenotypic variation, individual methylation variants may. We performed marginal [24, 28] and stepwise [29] GWAS using methylation variants as fixed effects instead of SNPs. The results were then compared to those obtained using SNPs as fixed effects. Per the results above, we used only SNP-based kinship estimates to control for population structure confounding (which it does well, see S1 Fig).

**Fig 2:**
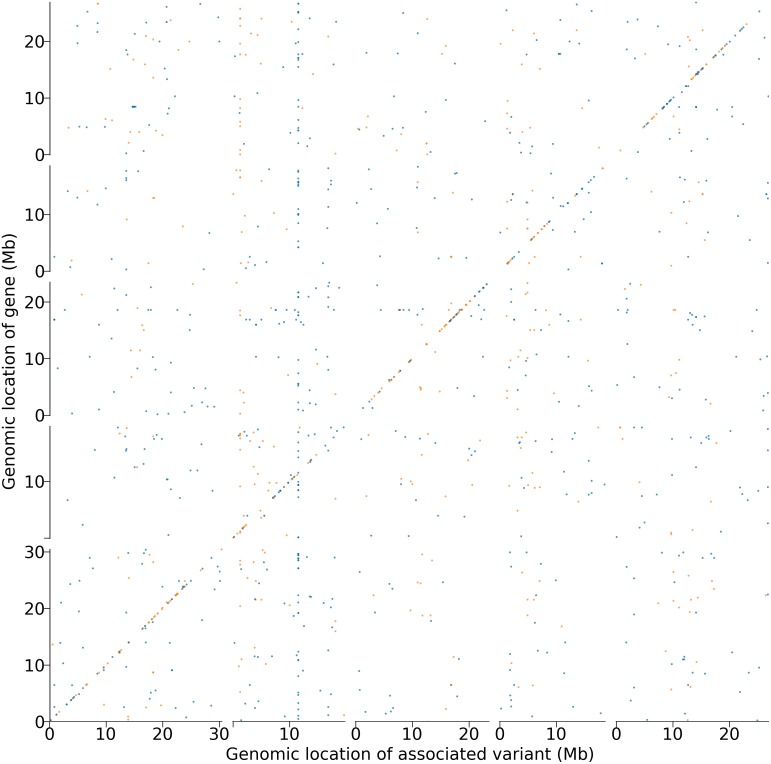
Genome-wide associations between expression and DNA methylation variation. For each gene, results are merged in 10 kb windows and a dot is plotted whenever the window contains at least one significantly associated variant (using a Bonferroni-corrected 5% threshold). Blue dots indicate CG-only methylation; orange C methylation.

A global view of significant methylation associations (Fig 2) shows an abundance of *cis*-associations with scattered instances of *trans*-associations, similar to what is observed for SNP-based associations (S2 Fig). A striking “hotspot” of putative *trans*-regulation was found near the center of chromosome 2, and corresponds to *AGO4*, a member of the Argonaute family involved in siRNA-mediated gene silencing [30, 31]. CG gene body methylation of *AGO4* (pattern in S3 Fig) is positively correlated with its expression, and expression of *AGO4* is strongly correlated with that of close to 70 other genes (seemingly unrelated; see S3 Table). Interestingly, no significantly associated SNPs were found, making this group of covarying genes detectable only using the methylation marker on *AGO4*.

While direct involvement of *AGO4* in transcriptional or post-transcriptional regulation is plausible [30–33], an alternative explanation is that all these genes are co-regulated, and that it is pure chance that methylation of *AGO4* is associated with its own expression, and therefore with the rest of the genes. Experiments to distinguish between these explanations are planned. In support of the latter explanation, there is very little little correspondence between SNP and methylation associations in *trans* (*cf.* S2 Fig and Fig 2), as would be expected if a large fraction of these associations were false positives.

**Fig 3:**
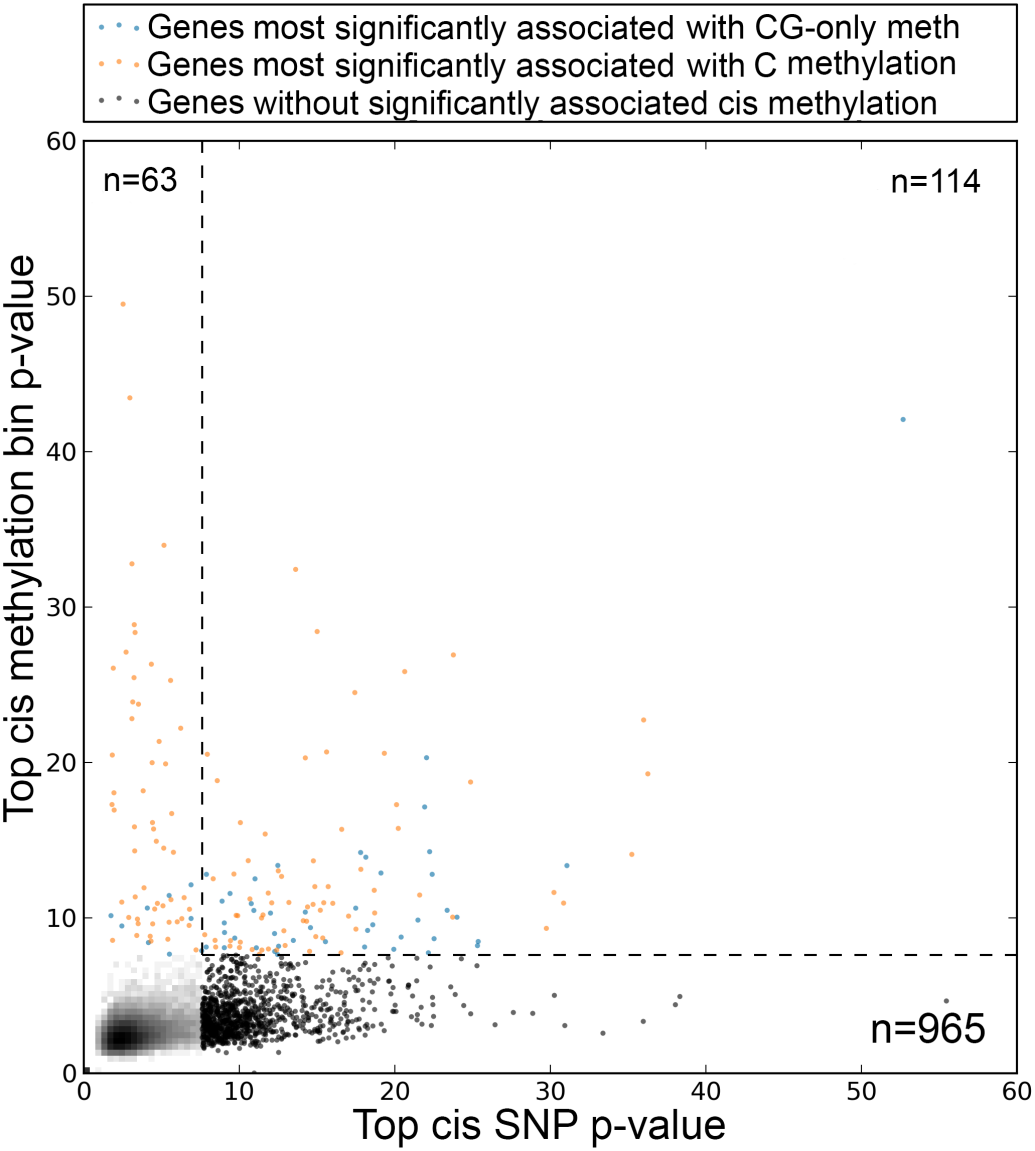
Top marginal associations in cis. Top methylation vs top SNP association with expression. The most significant p-value of association with 50 kb of the TSS for each gene is considered. Individual dots are shown for p-values less than a combined Bonferroni threshold of 10^−7.59^.

For the rest of the paper, we instead focus on *cis* effects, which are demonstrably real. Based on the over-representation of local (i.e., *cis*) vs. global (i.e., *trans*) effects, *cis*-methylation associations have a false-positive rate of less than 0.5% (Methods), and they also strongly overlap with SNP-associations. They are not nearly as common, however. As shown in Fig 3, there is at least an order of magnitude more SNP associations than there are methylation associations, and 114 of the 177 (64.4%) genes that have a significant methylation association also have a significant SNP association (S2 Table). This leaves 63 significant methylation associations without an accompanying SNP association. Most of these are not associated with any SNP even at less stringent significance thresholds (Fig 3), and the corresponding genes are thus candidates for being regulated epigenetically. It is worth that 55 of the 63 have C-methylation, suggesting the presence of transposable element.

**Fig 4:**
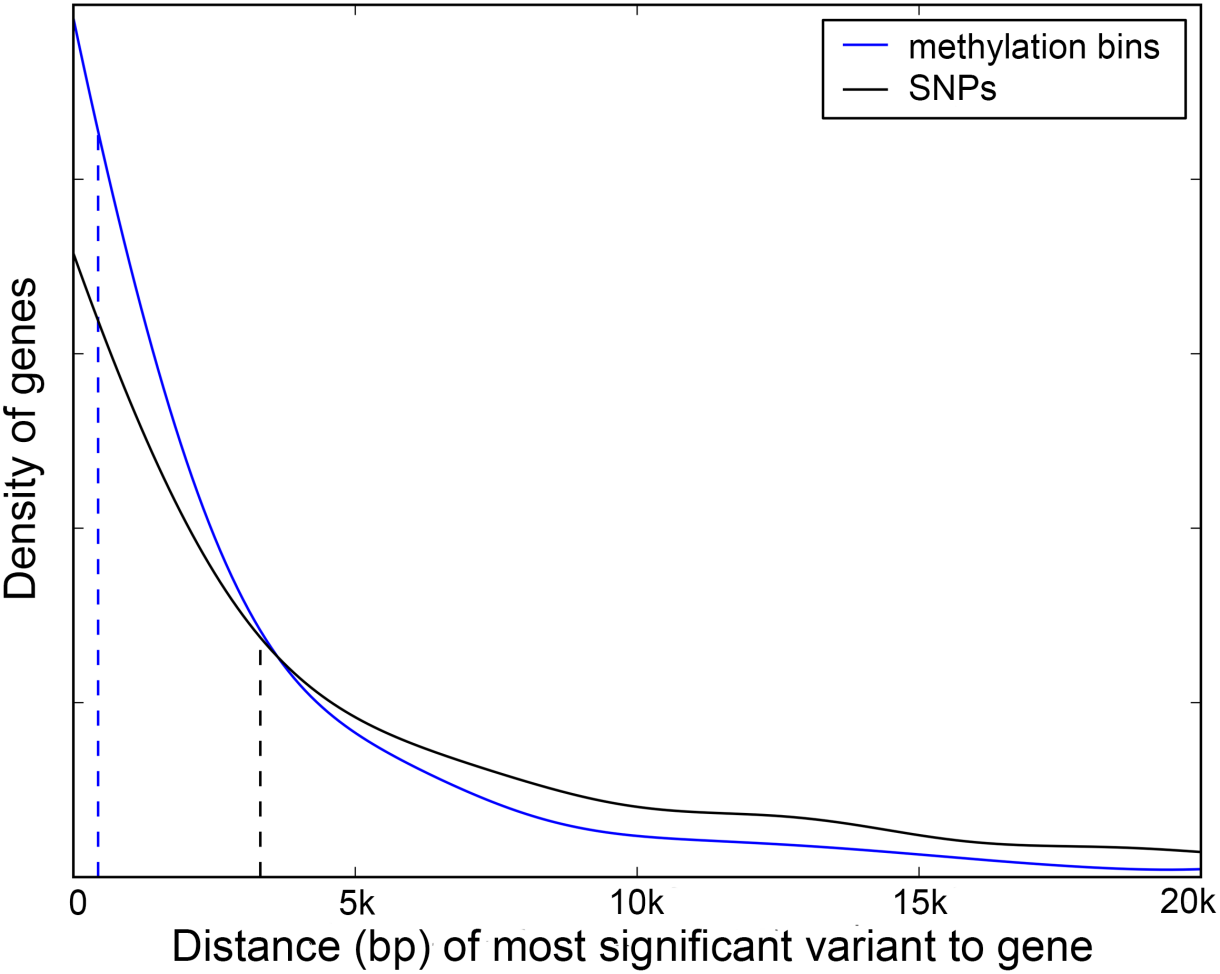
Distance of marginal associations in cis. Density plot for distribution of distances between most significant variant and transcribed part of gene, for SNPs and methylation variants, using only genes with significant association for both. The respective medians are shown with dashed lines.

An alternative explanation is that the methylation variation captures extensive allelic heterogeneity that is difficult to map [34, 35]. Allelic heterogeneity could also help explain another interesting finding, namely that methylation associations are typically closer to the gene of interest than are SNP associations when both are found (Fig 4). Such behavior is expected if the most significant SNP is a “tag” SNP that serves as a proxy for multiple underlying causal variants [34, 35].

### Additional variance explained by cis methylation

In order to capture additional effects of *cis* methylation more accurately, we used a nested model in which we first estimate genetic effects with a combination of random effect terms (based on local as well as global genetic similarity matrices [27]) and stepwise fixed-effect cofactors for remaining large effect SNPs, then capture any remaining methylation effects as stepwise fixed effects (See Methods for details).

**Fig 5:**
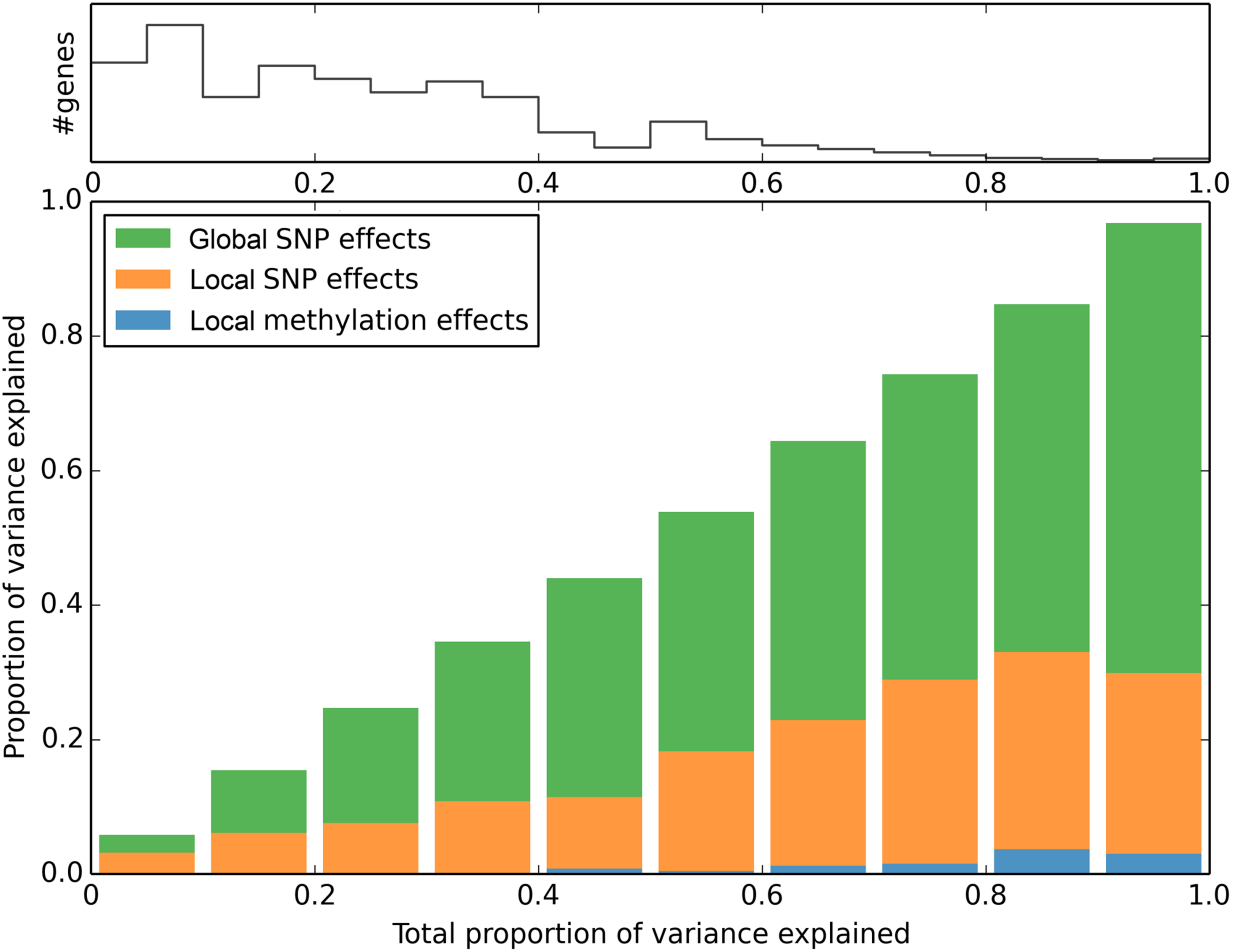
Partition of variance explained by local (*cis*) and global (*trans*) SNP effects, and by cis methylation effects for all expression traits. Traits are binned by the total variance explained, colored bars showing the average partitioning of the variance in each bin. The number of traits/genes in each bin is shown in the density plot on top.

Across genes, almost all heritable expression variation is due to genetic effects, with *cis*-methylation explaining only a small additional fraction of the variance (Fig 5). Nonetheless, the contribution is significant in a small number of cases. Using a Bonferroni-threshold based solely on methylation bins, we detected 212 significant associations between expression and DNA methylation. Of these, 64 remain significant after taking *cis*-SNP effects into account, 46 of which were already identified as having only *cis*-methylation in the previous section (S2 Table). Using an expanded data set that includes more genes for which a high proportion of individuals had no detectable expression (potentially due to epigenetic silencing), the corresponding counts are 397 and 148, respectively. Among the genes identified in this extended data set is *QQS*, a gene involved with starch metabolism which has been shown to be epigenetic regulated (albeit it in a different population) [36]. The genes with methylation associations span very diverse biological processes, but we find a significant enrichment for defense genes (p = 1.2e-06, FDR = 0.001; see Methods).

### Testing causality

That methylation is correlated with expression is clear, but whether there is a causal relationship, and, if so, in which direction it goes, is not. Transposon methylation is generally considered causally repressive in normal tissues, because disrupting methylation experimentally indeed often leads to transposon reactivation. However, little is known about gene body methylation, which is sometimes considered a consequence of transcriptional activity rather than a cause [37, 38]. Because non-disruptive methods to change DNA methylation experimentally are not available, this has been a difficult question to answer directly, but several attempts have been made using statistical causal models [16, 20], indirect inference with positional information [18], or stress induced changes [39]. We took the first approach, using a Bayesian network model-selection framework. A major challenge in our setting is the strong contribution of polygenic factors, even for relatively simple traits like expression. We explicitly included these factors in our models using a novel Bayes’ factor approach that expands upon existing methods [40–42].

**Fig 6:**
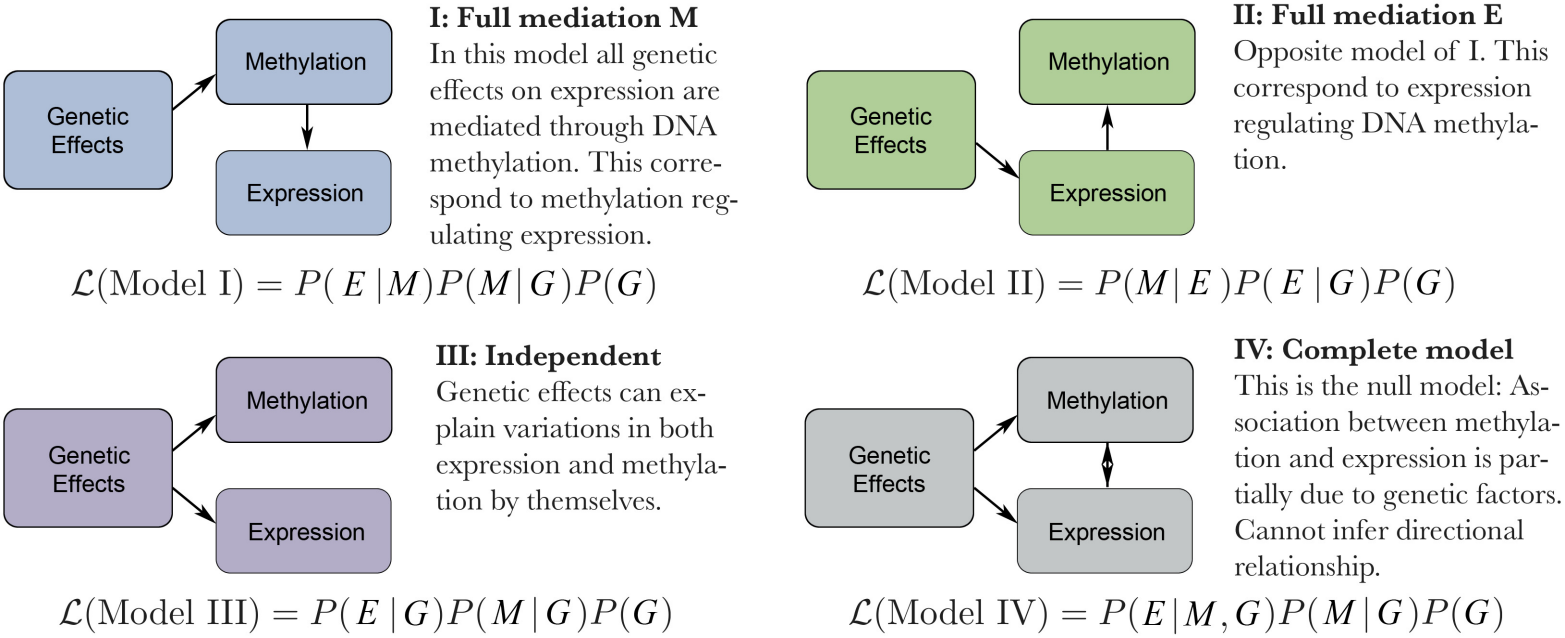
Description of causal models. Here L denote the likelihood of each model given data at each gene and *P* is the probability or conditional probability of individual measurements, *M* is the DNA methylation level and *E* is the expression level. Genetic effect include both fixed and random effects. For details see Methods.

We consider a total of four possible causal relationships between genetic variation, methylation variation, and expression variation (Fig 6). We are most interested in comparing the case where methylation regulates expression by mediating all genetic effects (Model I) to the case where the opposite is true (Model II), but we also consider the possibility that genetic variation affects both methylation and expression independently (Model III), and a “full model” where genetic variation affects both methylation and expression, which are also allowed to affect each other.

**Fig 7:**
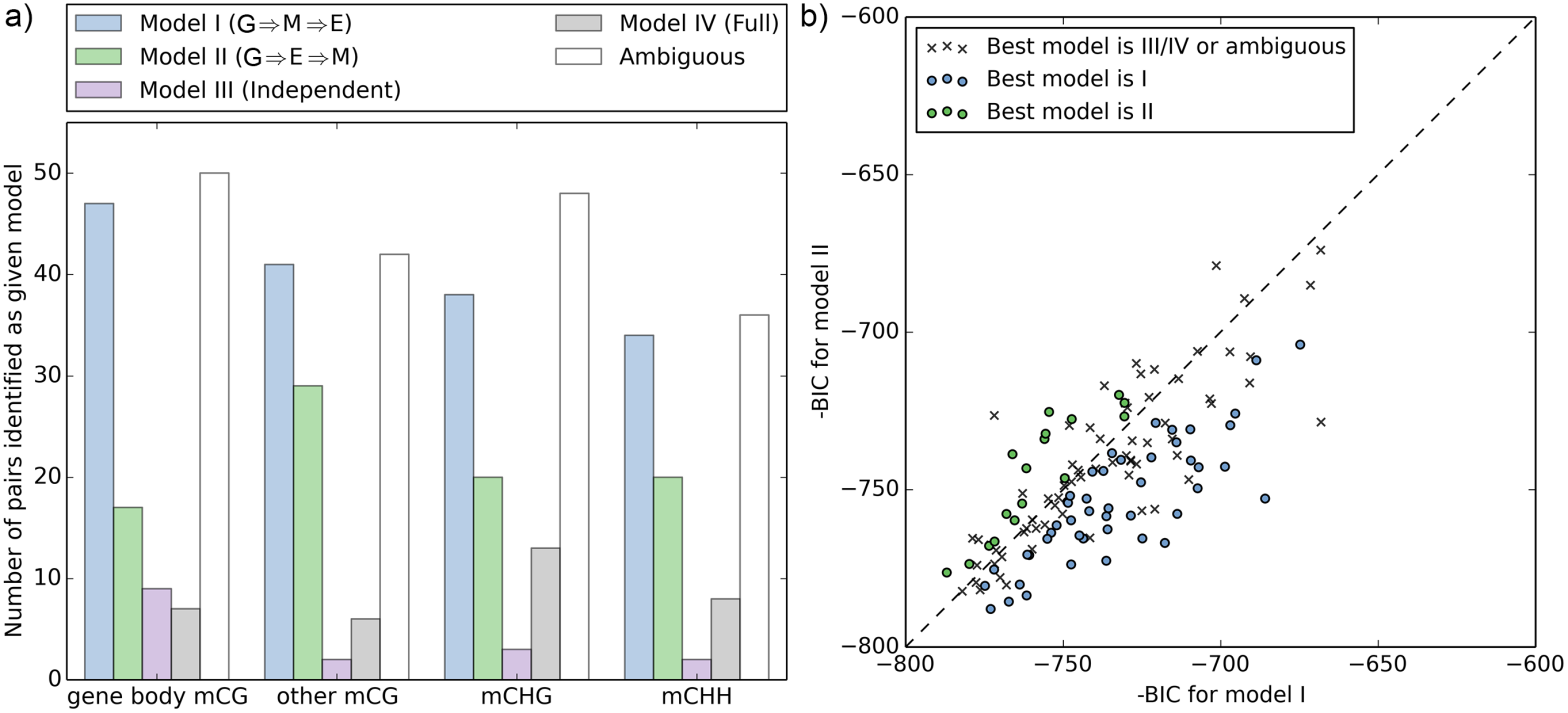
Comparison of causal models for methylation and expression variation. a) The number of genes identified as fitting a particular causal model (I-IV) significantly better, or as ambiguous (BIC difference between best and second best smaller than 3). b) BIC comparison for Models I and II, for GBM. Lower values correspond to a better fit to the model in question. For other types of methylations (e.g. CHG methylation), see S6 Fig.

For the 297 genes with significant associations among methylation, expression and SNPs, we calculated the likelihood for each of the four models and compared them using the Bayesian Information Criterion (BIC). For most genes, Model I is a better fit than Model II, although the difference is often not significant (Fig 7). This suggests that DNA methylation is affecting expression rather than the other way around, for CG-only as well as C methylation. However, whereas the inverse-normal transformation used in this paper seemed to produce more reliable GWAS results (see Methods), it may dampen effects in our causal model and cause bias. We therefore repeated the causality tests using untransformed versions of expression and DNA methylation data. The likelihood for all models increased, as expected given the removal of the dampening effect, be we also found a much stronger support for Model III (S7 Fig). Thus, while the relationship between Model I and II remained, suggesting that methylation is more likely to affect transcription directly than the other way around, the best-fitting model with untransformed data is one in which both methylation and transcription are caused by genetic variation without necessarily affecting each other. Statistics alone is unlikely to resolve this issue.

## Discussion

There is currently great excitement about the potential role of epigenetics in complex trait variation, both as a regulatory and as an inheritance mechanism. That an important role is in principle possible is not in doubt [2, 5], but there is almost no information on whether it actually matters in practice. This is a clearly a quantitative question and the answer will not be the same for all traits and populations.

In this paper, we focus on whether knowing the epigenome (in the form of DNA methylation variation) improves our understanding of expression variation in *A. thaliana* leaves. The general answer is: Only marginally (Fig 5). In terms of overall heritability, the genome-wide pattern of methylation polymorphism does not explain anything beyond the genome-wide pattern of SNP polymorphism, and while over a thousand expression traits have significant SNP associations, only about a hundred have associations with methylation variation, and most of these are also associated with SNPs. Indeed, no more than about sixty show evidence of a significant methylation association once SNP effects have been taken into account. Thus, although there are numerous caveats to our results (limited sample size, limited technology for measuring both expression and methylation, uncertainty about how to quantify methylation variation, *etc.*), our overall conclusion is that the effects of methylation variation are marginal relative to those of genetic variation. However, this does not mean that knowing the methylation variation is pointless. One interesting finding is that, for expression traits with both methylation and SNP associations, the former are often physically closer to the gene being expressed than the latter (Fig 4). This could be because the most highly associated SNPs are in fact just tagging multiple underlying causal SNPs [34, 43], and suggests methylation polymorphism could help with fine-mapping especially in a study with larger sample size. Equally importantly, we do find a small number of genes with clear evidence for epigenetic regulation, including several with no significant *cis*-SNP associations. These merit further investigation. The same is true for the minority of genes in which promoter methylation is positively rather than negatively correlated with expression (Fig 1). It should again be emphasized that our definition of methylation variation (average methylation in windows for different methylation contexts) is rather crude, and that it may be possible to define more biologically relevant statistics.

Finally, we address the issue of causality. In particular for gene body methylation, is debated whether the observed correlation is between methylation and expression is cause, effect, both, or neither [16, 20]. While we find support for methylation variation being a direct cause of expression variation, and perhaps even stronger support for both types of variation being influenced by genetics independently, our main conclusion is that is a question that will require direct experimental evidence to answer.

## Methods

### Data filtering and transformation

We used previously published polymorphism [44] and transcriptome/methylome data [7], which are available via the NHI Gene Expression Omnibus (GSE54292, GSE54680, GSE65685, GSE66017) and from the 1001 Genomes Project website. For the transcriptome/methylome data, only the data from the 10°C sexperiment were used. More details about growing conditions, tissues used and sequencing pipelines can be found in the relevant papers. In order to reduce the number of false associations while maintaining reasonable sensitivity, the three dataset were processed as follows.

#### SNPs

We used previously published SNPs [44], but removed all variants for which the minor allele frequency was less than 0.05. Given our limited sample size, we find that we have little power to detect effects from these rarer SNPs, while they produce more false positives due to the sensitivity of parametric linear regression to outliers.

#### Expression

We used both the filtered as well as raw rpkm values from RNAseq data in [7]. In addition, all genes whose mean expression level is lower than 3 after Anscombe transformation are removed. A minimum coefficient of variation of 0.05 is demanded for the remaining set so that only genes showing some variable expression are kept. Expression used is measured on a per gene level.

#### Methylation bins

We compared various encodings of DNA methylation data, ranging from binary individual data to gene averages. We choose to present all analysis performed on average of cytosine methylation level over all cytosine sites in 200-bp overlapping bins, which is much less noisy than individual methylation sites but retains a reasonable amount of fine level signal. We find varying bins both inside and outside of genes.

As mentioned in the main text, we divided those variants bins into four classes: CG where no non-CG methylation is present, CG where there is non-CG methylation present, CHG and CHH methylation. The binned methylation variants are computed separately as mCG/CG, mCHG/CHG, mCHH/CHH, and CG bins are separated into CG-only and CG in C bins by absence or presence of CHG methylation in the same 200 bp window.

Similar to expression levels, all methylation bins were filtered for a minimum coefficient of variation of 0.05. We also devised a new filter analogous to the minor allele frequency filter for SNPs but extended to quantitative data: methylation levels were normalized to *μ* = 0 and *σ*^2^ = 1, and the sum of squared distance of the 5 furthest values are calculated. Is this number is greater than 75, the methylation bin was eliminated from the analysis.

#### Inverse normal transformation for expression and DNA methylation

Despite stringent filtering and initial transformation, the remaining expression and DNA methylation data often diverge heavily from an expected normal distribution. In order to present only the most reliable associations, we also performed inverse normal transformation on both, effectively only keeping the rank information. This transformation was applied in all association studies involving marginal effects.

### Detecting genome-wide DNA methylation effects

We extended the SNP-based heritability models to include DNA methylation variants, which are similarly considered to follow independent and identically distributed Gaussian distribution, but with scale parameter $\sigma_{m}^{2}$. The structure of such effect is dictated by the “epigenetic similarity matrix” *K*_*M*_, calculated analogously to the SNP based genetic relatedness/similarity matrices [22]. We then perform likelihood ratio test between a model that include this epigenetic term and one that does not:

$$Y∼N(\mu ,K_{S}\sigma_{S}^{2}+K_{M}\sigma_{M}^{2}+\sigma_{\epsilon }^{2}),$$ (1)

$$Y∼N(\mu ,K_{S}\sigma_{S}^{2}+\sigma_{\epsilon }^{2}).$$ (2)

### Marginal and stepwise association mapping

GWAS using both genotype data and DNA methylation bins was performed with linear mixed models (as implemented in mixmogam: https://github.com/bvilhjal/mixmogam) to correct for population structure. The model used was

$$Y∼N(\mu +X\beta ,K_{s}\sigma_{s}^{2}+I\sigma_{e}^{2}),$$ (3)

where *Y* is the vector of phenotypes, *X* is a single vector of SNP or methylation bins, and the *β*’s correspond to allelic effect sizes. ***K***_*s*_ and $\sigma_{s}^{2}$ are again the genetic related matrix and its corresponding random effect size, while $\sigma_{e}^{2}$ is the residual variance due to unexplained environment or noise.

Marginal F-statistics were calculated as in ordinary linear model after rotating the phenotype *Y* and *X* by ${\left(\Lambda \delta +I\right)}^{-\frac{1}{2}}Q^{T}$, where *Q*Λ*Q*^*T*^ is the spectral decomposition of the symmetric relatedness matrix K and *δ* is the ratio between *σ*_*s*_ and *σ*_*e*_. To simplify calculations, we used the same approximation as in EMMAX [24], i.e., we only calculate the ratio *δ* once for the null model without fixed effects. The significance level (p-value) is then obtained by F-tests for SNPs and methylation bins of all contexts.

A direct extension of the marginal model is to include large effects as cofactors. This is accomplished in the forward stepwise mixed model [29] which result in a final model as

$$Y∼N(\mu +\sum_{i}^{n_{S}}X_{S,i}\beta_{S,i}+\sum_{j}^{n_{M}}X_{M,j}\beta_{M,j},K_{S}\sigma_{S}^{2}+I\sigma_{\epsilon }^{2}),$$ (4)

where *X*_*s*_ and *β*_*s*_ are SNP vectors and their respective effects, whiel *X*_*M*_ and *β*_*M*_ are methylation bin vectors and effects. At each step, the top marginal variant is added to *X*_*S*_ until no variants remains significant at Bonferroni threshold.

### Estimating false discovery rate of *cis* associations

We can derive a conservative upper bound for the false discovery rate for our *cis* associations, defined for each expression trait as everything less than 20 kb away from either end of the gene, by considering the over-representation of associations in *cis* compared to in *trans*, and assuming that all the latter are false. This is similar to what was previously done for candidate gene lists [34].

### Mapping methylation effect on top of SNP effects

We perform a association study that explicitly compare variance explained by DNA alone versus DNA methylation and DNA together. We try to capture large effect loci, effects due to allelic heterogeneity as well as background trans effects by using a linear mixed models that include an additional variance component for cis SNPs. In particular, the local equivalent of the global relatedness term is included which would capture most of cis effects from one or more (heterogeneous) loci. The full models are:

$$\text{genetics}:\;Y∼N(\mu +X_{S}\beta_{S},K_{S}\sigma_{S}^{2}+K_{l}\sigma_{l}^{2}+I\sigma_{\epsilon }^{2}),$$ (5)

$$\text{genetics}+\text{methylation:}\;Y∼N(\mu +X_{S}\beta_{S}+X_{M}\beta_{M},K_{S}\sigma_{S}^{2}+K_{l}\sigma_{l}^{2}+I\sigma_{\epsilon }^{2}),$$ (6)

where ***K***_*l*_ and $\sigma_{l}^{2}$ are the cis SNP kinship and its effect. We do not include a global methylation kinship since that has been found to exert no influence in most cases.

### Gene ontology enrichment analysis

We used the web tool AgriGo (http://bioinfo.cau.edu.cn/agriGO/) [45] to find functional categories that is significantly enriched in the subset of *cis*-methylation associated genes.

### Data preparation for causal analysis

We prepared the following set of data for use in causal structure analysis, with the goal being to identify pairs of associated expression/methylation that also shows evidence of being associated with the same genetic factors. We first correlated expression level with all cis methylation bins that is within the gene or within 2000 bp of the transcription start site. If any bin is correlated with a *r*^2^ greater than 0.2, the pair of expression and the highest correlated bin is added to a testing pool. From this pool, mixed model GWAS is performed on each pair of expression/methylation, and any pair that does not:

1. share associated SNP at the Bonferroni threshold for *trans*-SNPs (defined as greater than 50 kb away) or at 10^−5^ for *cis*-SNPs, or;
2. have the genetic kinship component explain at least 5% of variance,

is filtered out. This results in a final set of data from 297 genes.

### Causal analysis

We build upon earlier statistical framework [40–42] for causal analysis. Our methods try to infer causal relationship between three variables: genetic factors (G), or more precisely DNA sequence; DNA methylation (M); and phenotypic trait, in this context mostly referring to expression traits (E). Among these, it is assumed that genetic factors (G) are not subject to influences from the other factors. This is not true in general due to effects of selection and mutation rates on DNA sequences, but these effects are negligible for data collected in this study that are at most several generations apart. We thus reduce to the four possible scenarios in Fig 6.

Here our goal is to distinguish between the four potential models considered. We base our selection on Bayesian information criteria that are calculated from maximum likelihood of the respective models. These likelihoods are calculated as:

$$\begin{array}{ll} \text{Model I: }L(M_{1}|g,m,e) & =p(e|m)p(m|g)p\left(g\right)\\ \text{Model II: }L(M_{2}|g,m,e) & =p(m|e)p(e|g)p\left(g\right)\\ \text{Model III: }L(M_{3}|g,m,e) & =p(e|g)p(m|g)p\left(g\right)\\ \text{Model IV: }L(M_{4}|g,m,e) & =p(e|m,g)p(m|g)p\left(g\right)\\ & =p(m|e,g)p(e|g)p\left(g\right) \end{array}$$ (7)

In cases where M is confined to one observation per individual like expression levels, the relationship between E and M are considered linear with Gaussian noise:

$$\begin{array}{c} p\left(e|m\right)∼N\left(\mu_{E}+m\beta_{M},I\sigma_{\epsilon_{E}}^{2}\right){|}_{m}\\ p\left(m|e\right)∼N\left(\mu_{M}+e\beta_{E},I\sigma_{\epsilon_{M}}^{2}\right){|}_{e} \end{array}$$ (8)

Whereas the distribution involving genotype would contain both fixed terms for large effects as well as random terms for genetic background:

$$\begin{array}{ll} p(e|g) & ∼N\left(\mu_{E}+X\beta_{X,M},K_{S}\sigma_{S,E}^{2}+I\sigma_{\epsilon_{E}}^{2}\right){|}_{g}\\ p(m|g) & ∼N\left(\mu_{M}+X\beta_{X,E},K_{S}\sigma_{S,M}^{2}+I\sigma_{\epsilon_{M}}^{2}\right){|}_{g}\\ p(e|m,g) & ∼N\left(\mu_{E}+X\beta_{X,M}+m\beta_{M},K_{S}\sigma_{S,E}^{2}+I\sigma_{\epsilon_{E}}^{2}\right){|}_{m,g}\\ p(m|e,g) & ∼N\left(\mu_{M}+X\beta_{X,E}+e\beta_{E},K_{S}\sigma_{S,M}^{2}+I\sigma_{\epsilon_{M}}^{2}\right){|}_{e,g} \end{array}$$ (9)

The maximum likelihood of Eq 8 is calculated by least square estimate of *β*s, while those of Eq 9 are found by numerical method implemented in mixmogam. Since we are interested in the likelihood rather than estimates of the variance parameters, the ‘ML’ criteria (instead of restricted ML) is chosen as the optimization criteria for the latter. After we obtain the maximum log likelihood for each component, we sum them to obtain the overall log likelihood of the models minus a constant. Bayesian information criteria is chosen as our model selection criteria, corresponding to the fact that we already have all potential models chosen. It is calculated as:

$$\text{BIC}=-2\ln L_{i}+k_{i}\ln135,$$ (10)

where $L_{i}$ are the likelihood for models I-IV and *k*_*i*_ the corresponding number of free parameters.

### Simulation study for causal analysis

To investigate performance of our causal model, we performed a simulation study by generating pairs of traits using the our *A. thaliana* SNP dataset. Three sets of simulations are run:

1. Model I/II: M/E is the summation of 0–1 large effect (*Xβ* term), 10000 small effects ($K_{S}\sigma_{S}^{2}$ term), and a Gaussian error; Trait E/M is M/E plus another Gaussian error.
2. Model III: Trait E and M are both sum of 0–1 shared large effect, a combination of shared and private small effects totaling 10000, and a Gaussian error.
3. Model IV: Similar to III, but one of the traits also contain a linear term of the other.

These effects are scaled to achieve various levels of heritability.

Based on the results, when the underlying model is I (II) or III, we can deduce the correct model most of the time. However, when the real model is IV, it is very hard to capture. These results are summarized in S9 Fig.

## Supporting Information

S1 Fig QQ plots of SNP and DNA methylation marginal association p-values. SNP-based kinship correction works for *trans* effects, and there is the expected inflation of *cis* p-values.
(TIF)

S2 Fig Genome-wide associations between expression and SNPs. Similar as the figure for methylation bins, results are merged in 10 kb windows and a dot is plotted whenever the window contains at least one significantly associated variant. Here red is a SNP only peak whereas black means a bin where SNP peak overlaps with methylation peak(s).
(TIF)

S3 Fig DNA methylation pattern around AGO4 across accessions. Deeper blue means higher level of methylation. Only CG methylation is plotted, since other types of methylation are not present. The green and blue horizontal lines are the transcription start and stop sites, respectively.
(PNG)

S4 Fig QQ plots of cis variant f-test p-values after removing various cis effects by including them as cofactors. a) cis SNP p-value distribution changes after adding SNP local relatedness term and other cofactors. b) cis DNA methylation p-value distribution changes after adding SNP and DNA methylation cofactors. Most SNP effects are accounted for by the SNP local relatedness/kinship term. The rest of the methylation effects are considered independent.
(TIF)

S5 Fig Additional cis methylation effects for genes with significant effects. Showing, for the genes with significant additional methylation effect, the fraction of variance explained by combined cis methylation bins and SNPs fixed terms versus SNPs alone.
(TIF)

S6 Fig BIC comparison for model I and II, for methylation in all contexts. a) CG gene body methylation (Same as figure in text). b) CG methylation in C methylation context. c) CHG methylation. d) CHH methylation.
(TIF)

S7 Fig Comparing between methylation being causative versus reactive to expression variation, untransformed data version. Model III (independent) is the most frequently assigned with untransformed data. However the relative evidence for Model I and II remain in the same direction.
(TIF)

S8 Fig BIC comparison for model I and II, for methylation in all contexts, untransformed data version. a) CG gene body methylation. b) CG methylation in C methylation context. c) CHG methylation. d) CHH methylation. Note that the y-scale is much larger than in S6 Fig, since likelihood for all models are higher with untransformed data.
(TIF)

S9 Fig Simulation study for causal analysis. On the y-axis is the model from which data is simulated from, while on x-axis the breakdown of predicted models is shown.
(TIF)

S1 Table Number of data records used to plot the distribution of pearson’s r for each bin in Fig 1. (TSV)

S2 Table Significant cis DNA methylation associations. The column “any cis SNP significant” is true when there is a cis SNP association that passes the 5% Bonferroni threshold of 10^−7.59^.
(TSV)

S3 Table Genes whose expression is associated with DNA methylation at AGO4. (TSV)

S4 Table GO enrichment for genes with cis DNA methylation associations. (TSV)
