# Supplementary figures and images for: Limited Contribution of DNA Methylation Variation to Expression Regulation in Arabidopsis thaliana
Source: PLoS Genet. 2016 Jul 11;12(7):e1006141. doi: 10.1371/journal.pgen.1006141 (PMC4939946; doi:10.1371/journal.pgen.1006141)

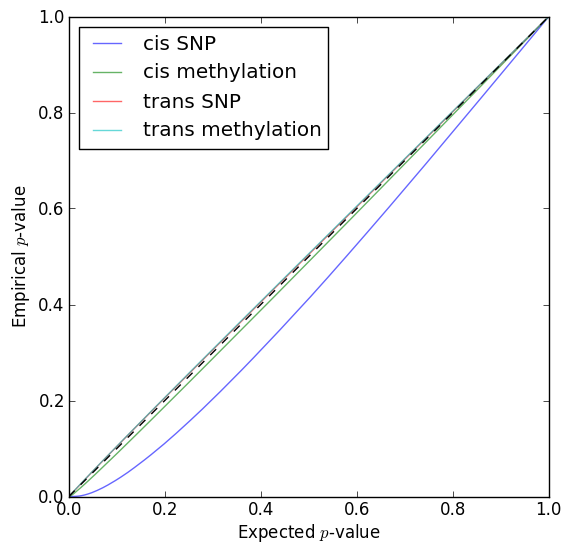

Supplement: S1 Fig — SNP-based kinship correction works for trans effects, and there is the expected inflation of cis p-values. (TIF) [file pgen.1006141.s001.tif]

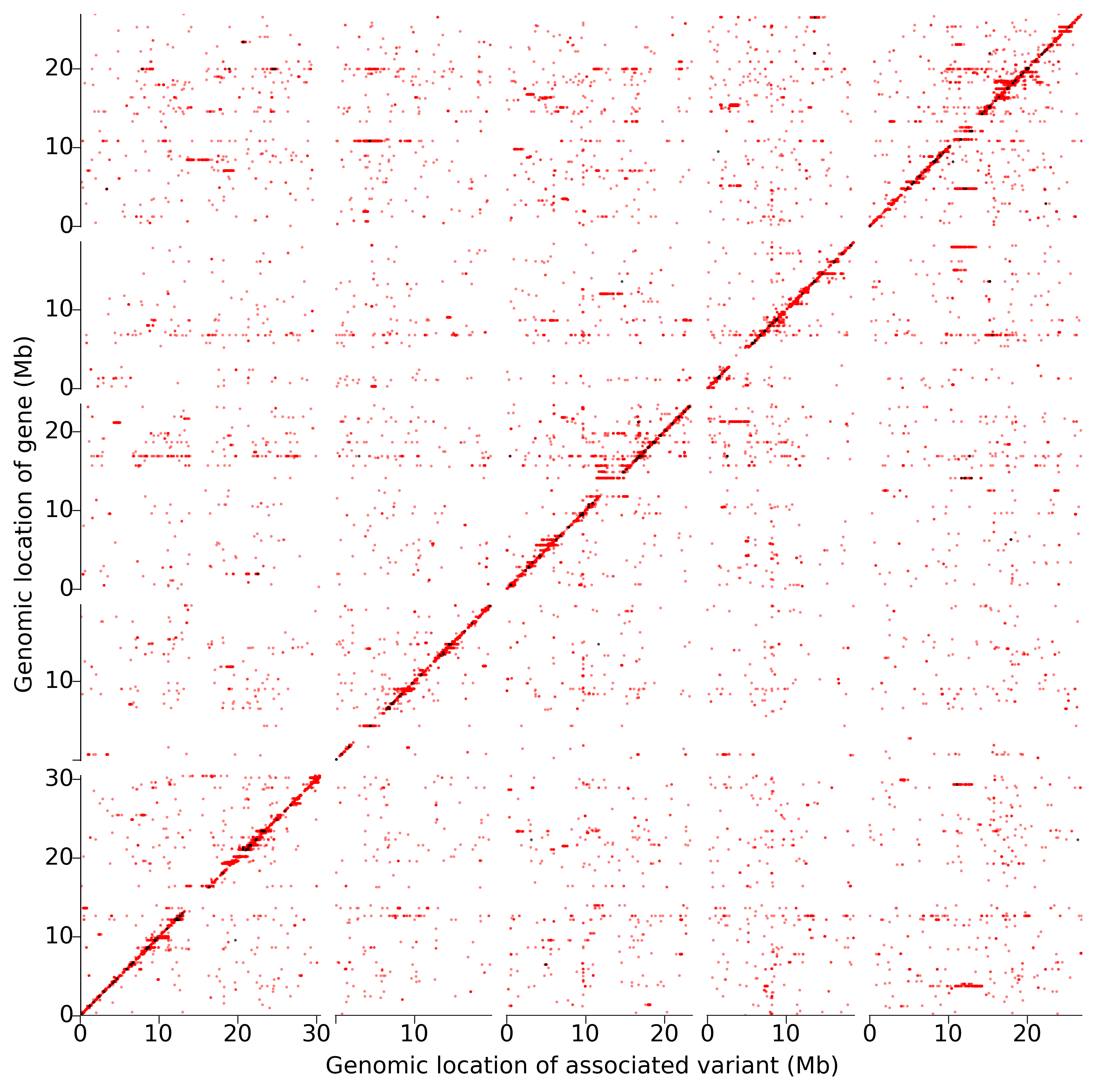

Supplement: S2 Fig — Similar as the figure for methylation bins, results are merged in 10 kb windows and a dot is plotted whenever the window contains at least one significantly associated variant. Here red is a SNP only peak whereas black means a bin where SNP peak overlaps with methylation peak(s). (TIF) [file pgen.1006141.s002.tif]

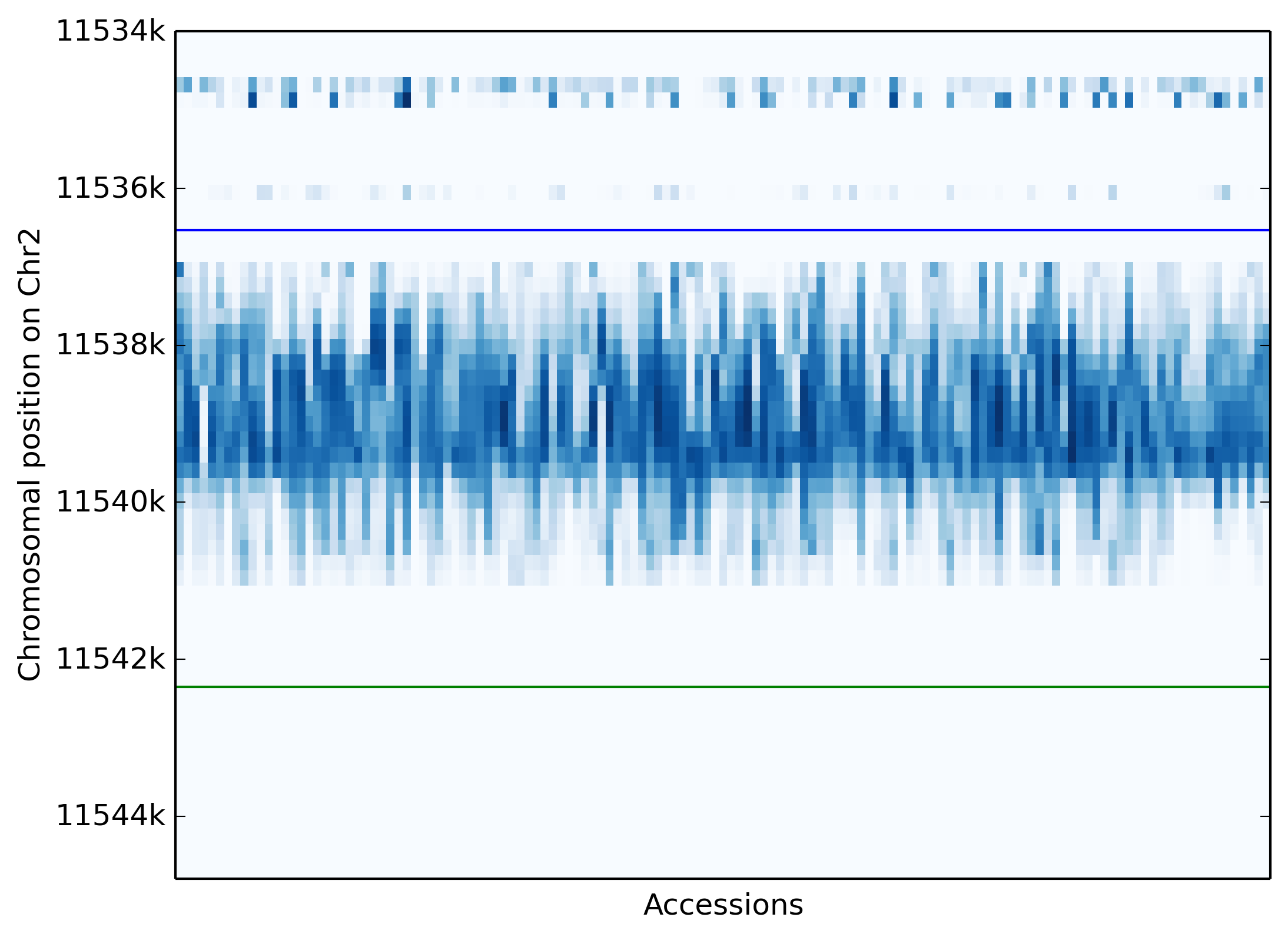

Supplement: S3 Fig — Deeper blue means higher level of methylation. Only CG methylation is plotted, since other types of methylation are not present. The green and blue horizontal lines are the transcription start and stop sites, respectively. (PNG) [file pgen.1006141.s003.png]

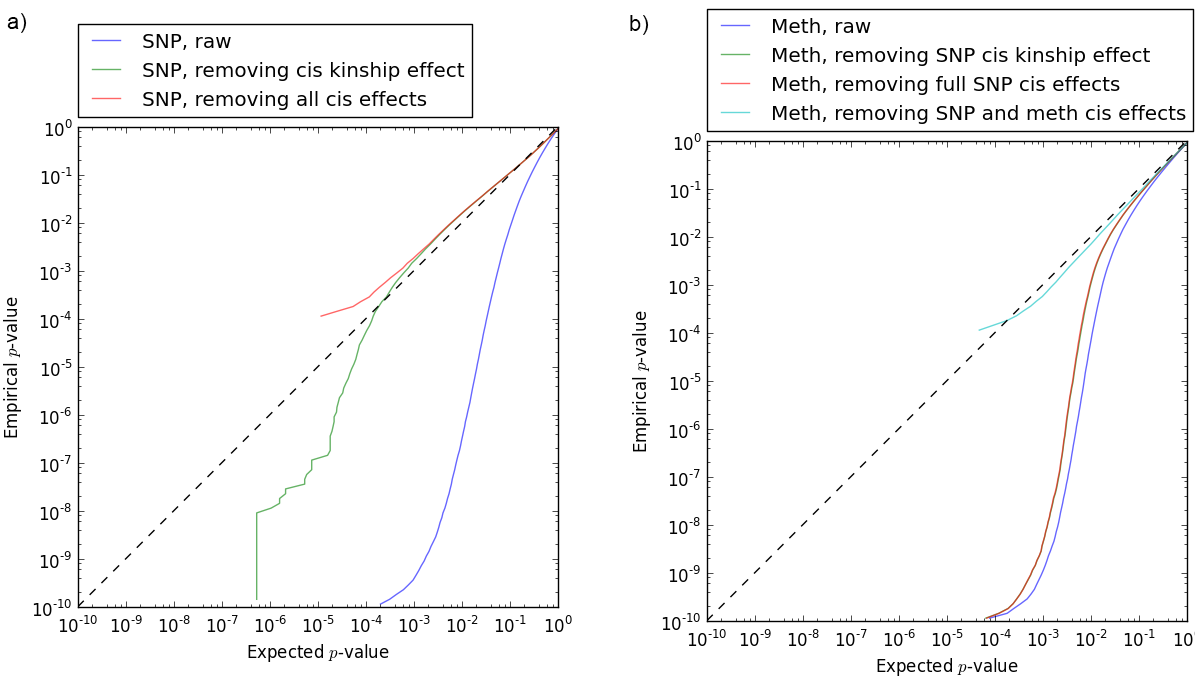

Supplement: S4 Fig — a) cis SNP p-value distribution changes after adding SNP local relatedness term and other cofactors. b) cis DNA methylation p-value distribution changes after adding SNP and DNA methylation cofactors. Most SNP effects are accounted for by the SNP local relatedness/kinship term. The rest of the methylation effects are considered independent. (TIF) [file pgen.1006141.s004.tif]

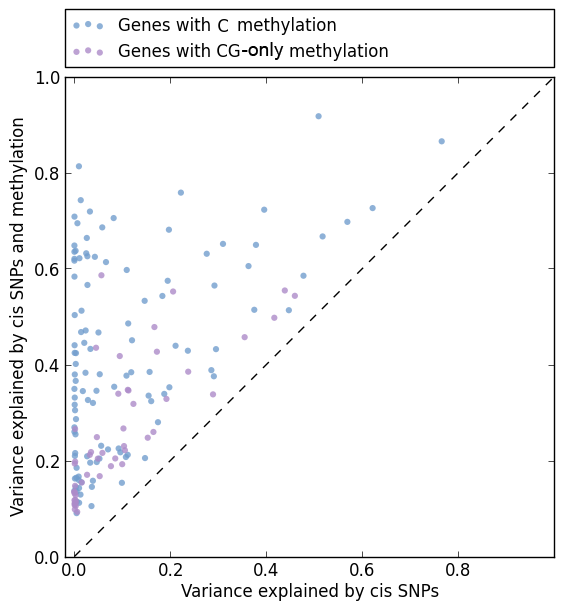

Supplement: S5 Fig — Showing, for the genes with significant additional methylation effect, the fraction of variance explained by combined cis methylation bins and SNPs fixed terms versus SNPs alone. (TIF) [file pgen.1006141.s005.tif]

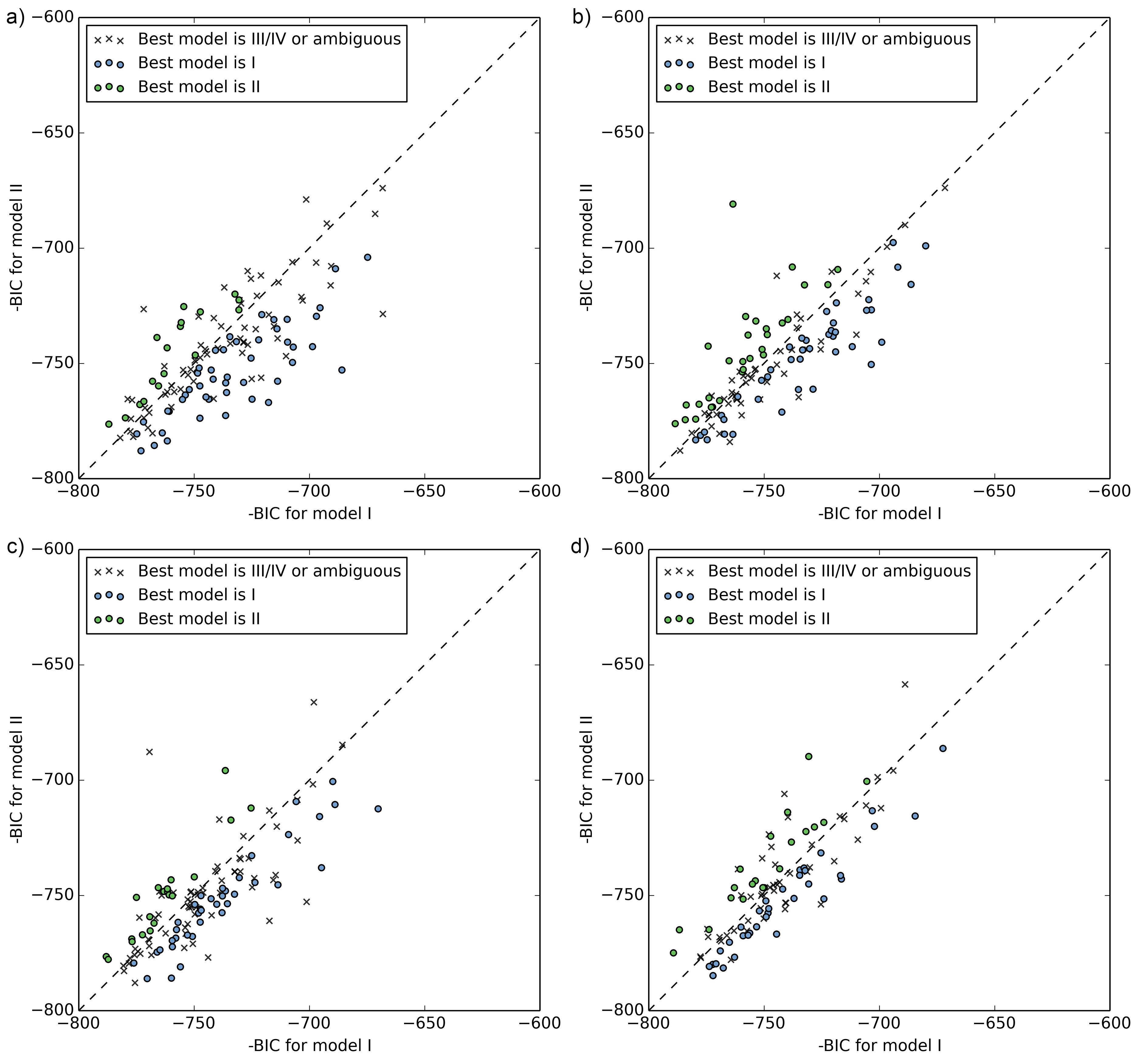

Supplement: S6 Fig — a) CG gene body methylation (Same as figure in text). b) CG methylation in C methylation context. c) CHG methylation. d) CHH methylation. (TIF) [file pgen.1006141.s006.tif]

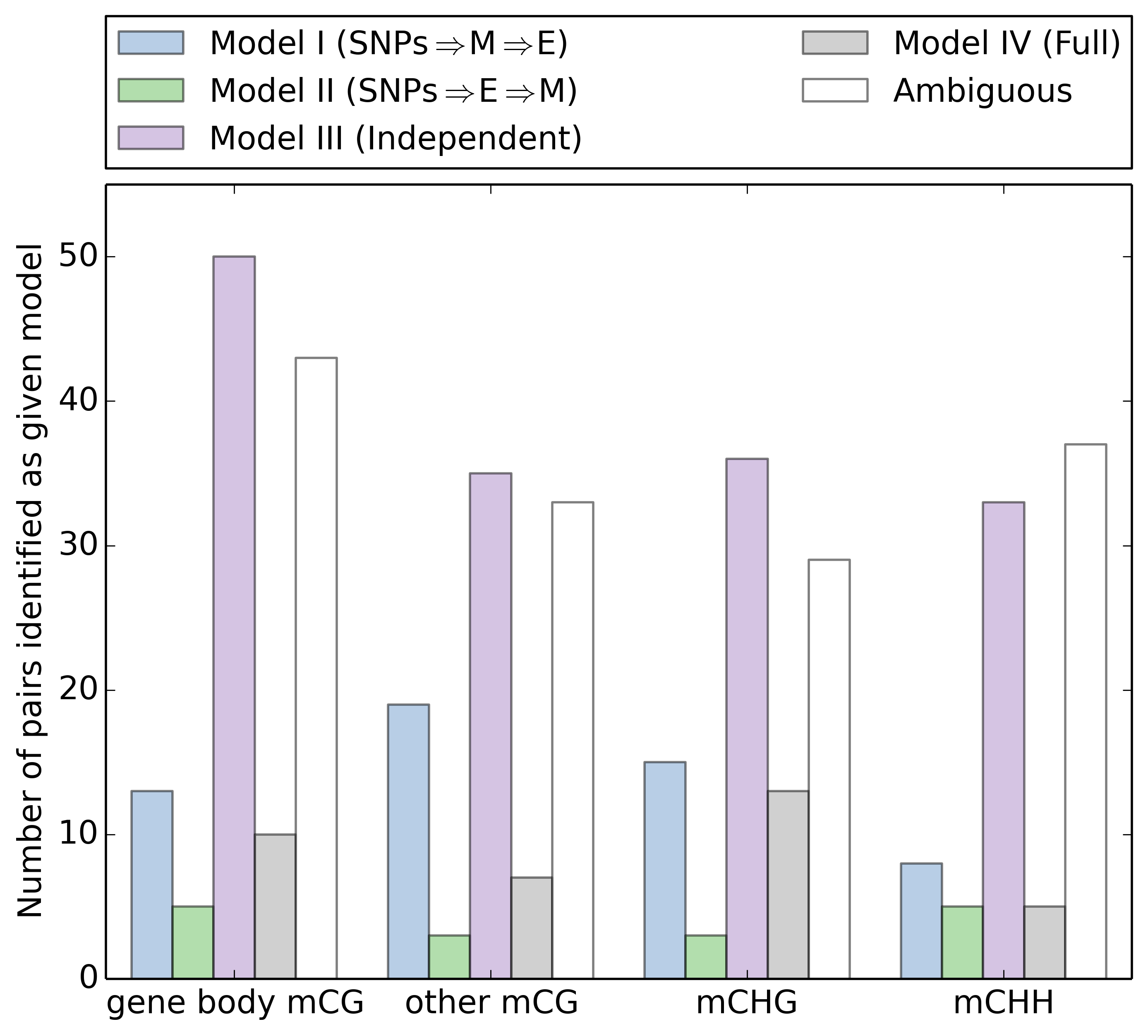

Supplement: S7 Fig — Model III (independent) is the most frequently assigned with untransformed data. However the relative evidence for Model I and II remain in the same direction. (TIF) [file pgen.1006141.s007.tif]

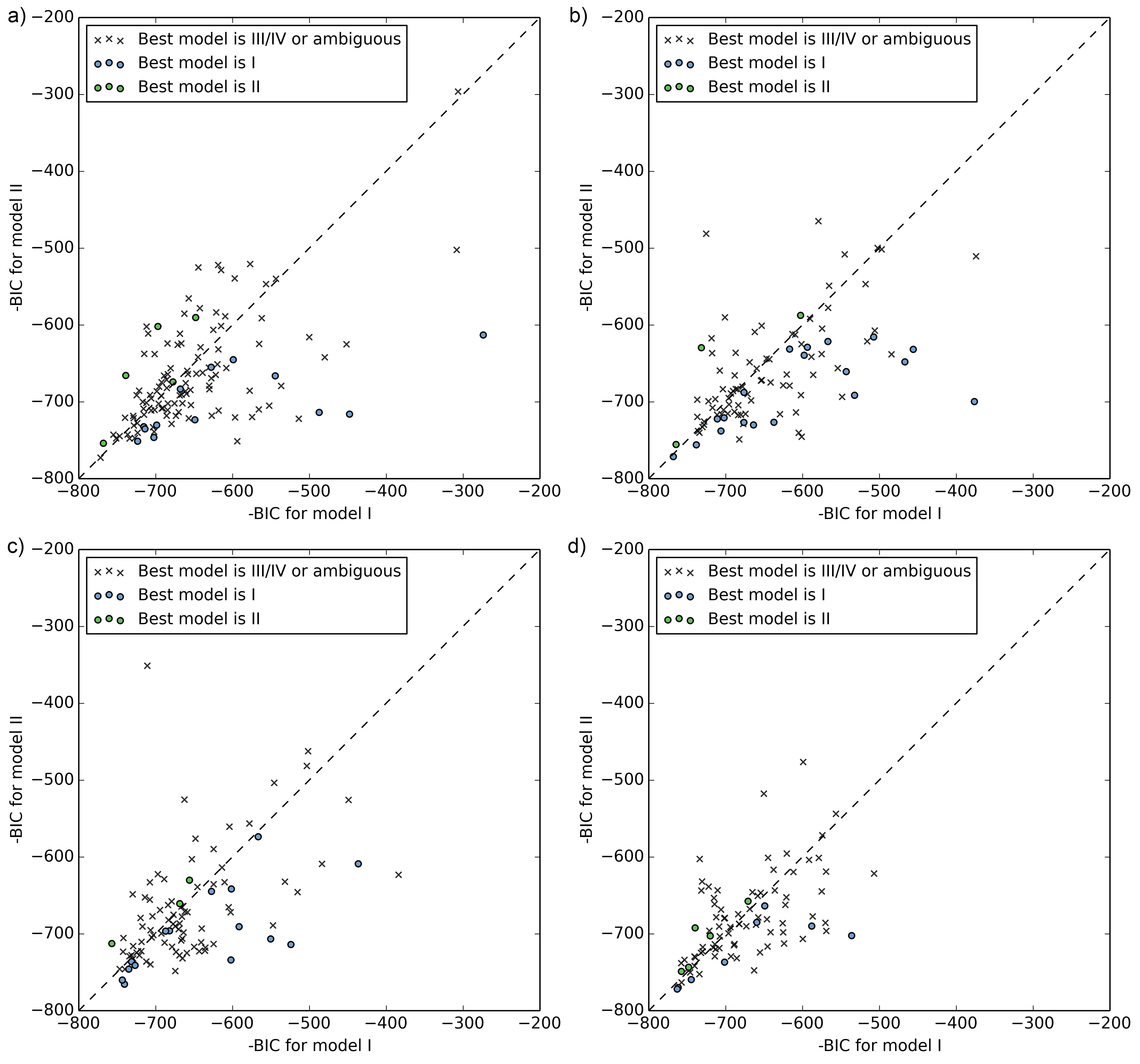

Supplement: S8 Fig — a) CG gene body methylation. b) CG methylation in C methylation context. c) CHG methylation. d) CHH methylation. Note that the y-scale is much larger than in S6 Fig, since likelihood for all models are higher with untransformed data. (TIF) [file pgen.1006141.s008.tif]

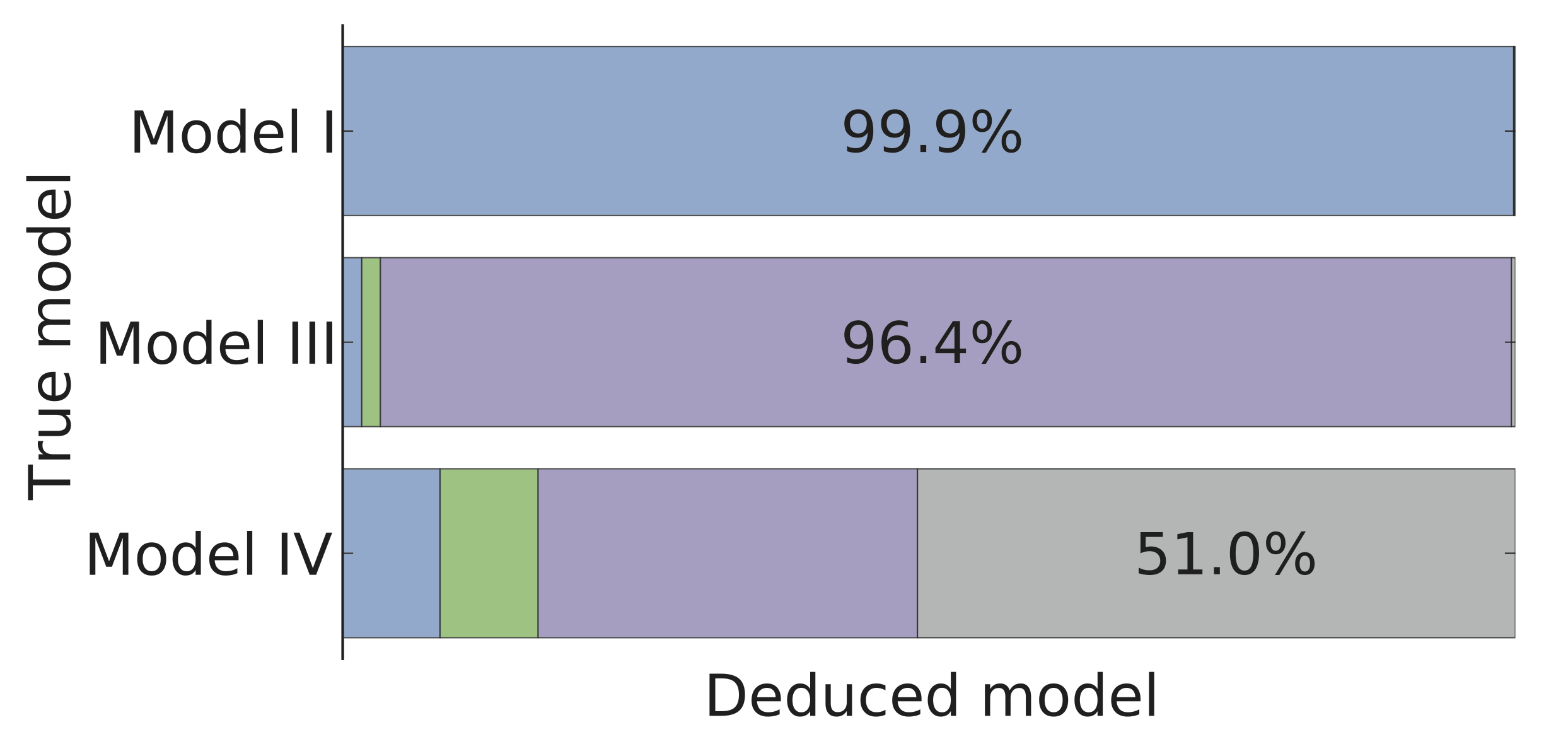

Supplement: S9 Fig — On the y-axis is the model from which data is simulated from, while on x-axis the breakdown of predicted models is shown. (TIF) [file pgen.1006141.s009.tif]
